# Interaction of Remdesivir with Clinically Relevant Hepatic Drug Uptake Transporters

**DOI:** 10.3390/pharmaceutics13030369

**Published:** 2021-03-10

**Authors:** Anne T. Nies, Jörg König, Ute Hofmann, Charlotte Kölz, Martin F. Fromm, Matthias Schwab

**Affiliations:** 1 Dr. Margarete Fischer-Bosch Institute of Clinical Pharmacology, 70376 Stuttgart, Germany; anne.nies@ikp-stuttgart.de (A.T.N.); ute.hofmann@ikp-stuttgart.de (U.H.); charlotte.koelz@ikp-stuttgart.de (C.K.); 2 University of Tuebingen, 72076 Tuebingen, Germany; 3 iFIT Cluster of Excellence (EXC2180) “Image Guided and Functionally Instructed Tumor Therapies”, University of Tuebingen, 72076 Tuebingen, Germany; 4 Institute of Experimental and Clinical Pharmacology and Toxicology, Friedrich-Alexander-Universität Erlangen-Nürnberg, 91054 Erlangen, Germany; joerg.koenig@fau.de (J.K.); martin.fromm@fau.de (M.F.F.); 5 Departments of Clinical Pharmacology, and of Pharmacy and Biochemistry, University of Tuebingen, 72076 Tuebingen, Germany

**Keywords:** remdesivir, drug transporters, OATP1B1, OATP1B3, OATP2B1, OCT1, inhibition, drug-drug interaction

## Abstract

Remdesivir has been approved for treatment of COVID-19 and shortens the time to recovery in hospitalized patients. Drug transporters removing remdesivir from the circulation may reduce efficacy of treatment by lowering its plasma levels. Information on the interaction of remdesivir with drug transporters is limited. We therefore assessed remdesivir as substrate and inhibitor of the clinically relevant hepatic drug uptake transporters organic anion transporting poly-peptide (OATP)-1B1 (*SLCO1B1*), its common genetic variants OATP1B1*1b, OATP1B1*5, OATP1B1*15, as well as OATP1B3 (*SLCO1B3*), OATP2B1 (*SLCO2B1*) and organic cation transporter (OCT)-1 (*SLC22A1*). Previously established transporter-overexpressing cells were used to measure (i) cellular remdesivir uptake and (ii) cellular uptake of transporter probe substrates in the presence of remdesivir. There was a high remdesivir uptake into vector-transfected control cells. Moderate, but statistically significant higher uptake was detected only for OATP1B1-, OATP1B1*1b and OATP1B1*15-expressing cells when compared with control cells at 5 µM. Remdesivir inhibited all investigated transporters at 10 µM and above. In conclusion, the low uptake rates suggest that OATP1B1 and its genetic variants, OATP1B3, OATP2B1 and OCT1 are not relevant for hepatocellular uptake of remdesivir in humans. Due to the rapid clearance of remdesivir, no clinically relevant transporter-mediated drug-drug interactions are expected.

## 1. Introduction

Remdesivir is a small molecule nucleoside analog inhibitor developed for treating diseases caused by RNA viruses such as Ebola virus [1]. As remdesivir also effectively inhibits severe acute respiratory syndrome coronavirus 2 (SARS-CoV-2) replication in vitro and in preclinical animal models of coronavirus disease 2019 (COVID-19), it was evaluated in several clinical trials [2]. Remdesivir (Veklury) is now the first Food and Drug Administration (FDA)-approved anti-viral agent to treat hospitalized COVID-19 patients [3,4]. It is a phosphoramidate prodrug that needs to be converted by esterases, phosphoramidases and kinases into its active nucleoside triphosphate analog inhibiting viral RNA-dependent RNA polymerase in target cells [1,2]. Remdesivir is administered intravenously because phosphoramidates are known to experience a significant first-pass effect [5] indicating a considerable uptake into the liver. Removal of remdesivir from the circulation may lead to subtherapeutic plasma concentrations and subsequently to reduced treatment efficacy. It is therefore important to investigate molecular mechanisms of hepatocellular remdesivir uptake.

Many studies have shown that uptake transporters in the sinusoidal membrane of hepatocytes, such as members of the organic anion transporting polypeptides (OATPs) family or organic cation transporters (OCTs), play an important role for drug absorption, distribution and effects [6,7,8,9,10]. The three OATPs expressed in hepatocytes, i.e., OATP1B1 (encoded by *SLCO1B1*), OATP1B3 (*SLCO1B3*) and OATP2B1 (*SLCO2B1*), mediate the uptake of a broad range of anionic endogenous compounds and drugs from blood [10,11]. Clinical studies underscore the major impact of common genetic variants in the *SLCO1B1* gene affecting OATP1B1 function (OATP1B1*1b, OATP1B1*5, OATP1B1*15) on plasma concentrations, therapeutic effects and adverse effects of many drugs including statins [11,12,13]. Moreover, the inhibition of hepatic OATPs by co-administered drugs may cause clinically-relevant drug-drug interactions [9,11,12].

Hepatic OCT1 (encoded by *SLC22A1*) is also an important determinant of drug distribution with consequences for drug exposure and response [7]. OCT1 transports various lipophilic cationic endogenous compounds and clinically-relevant drugs such as metformin, opioids and fenoterol from blood into hepatocytes [14,15,16]. Common genetic variants resulting in functional-deficient OCT1 have major impacts on drug pharmacokinetics and response as exemplified for fenoterol [16]. Similar to OATPs, clinically-relevant drug-drug interactions have been described [7,11].

Based on this acknowledged importance for the hepatocellular uptake of clinically-relevant drugs, OATP1B1, OATP1B3, OATP2B1 and OCT1 are requested by the regulatory authorities of drug approval, FDA and European Medicines Agency (EMA), to be studied with respect to their drug interaction potential [6,7,17]. Thus far, information on drug transporters involved in remdesivir uptake is limited to the information in the package insert provided for Veklury, in which OATP1B1, but not OATP1B3, was identified as a transporter of remdesivir [4].

We therefore tested the hypothesis that genetic variants of the clinically-relevant uptake transporter OATP1B1 affect cellular remdesivir uptake, which might have consequences on remdesivir pharmacokinetics. Secondly, we aimed to analyze whether it is a substrate of the additional important hepatic uptake transporters OATP2B1 and OCT1. Finally, we examined the inhibitor potential towards OATP1B1, OATP1B3, OATP2B1 and OCT1, which are frequently involved in drug-drug interactions.

## 2. Materials and Methods

### 2.1. Reagents

Remdesivir (purity > 99%) was from Selleck Chemicals (#S8932, Houston, Tx, USA, 10 mM stock solution in DMSO). 6,7-dimethyl-2,3-di(2-pyridyl) quinoxaline (QX; purity ≥ 97.5%) and all other chemicals were from Merck KGaA (Darmstadt, Germany). [^3^H]Sulfobromophthalein (14 Ci/mmol) (BSP) and [^14^C]metformin (107 mCi/mmol) were purchased from Hartmann Analytic GmbH (Braunschweig, Germany). [Estradiol-6,7-^3^H(N)]-17β-glucuronide (45.7 Ci/mmol) and [6,7-^3^H(N)]estrone sulfate (51.8 Ci/mmol) were from PerkinElmer (Boston, MA).

### 2.2. Cell Lines Stably Expressing Human Hepatic Uptake Transporters

Generation, cultivation and characterization of human embryonic kidney (HEK) cells stably expressing OATP1B1 refseq (=OATP1B1*1a), OATP1B1*1b, OATP1B1*5 and OATP1B1*15 [18], OATP1B3 [19], OATP2B1 [13] and OCT1 [20] have been described previously. OATP1B1 cells were cultivated in minimal essential medium (Life Technologies, Paisley, UK) and OATP1B3, OATP2B1 and OCT1 cells were cultivated in Dulbecco’s modified Eagle medium (Sigma, Darmstadt, Germany). Cultivation media were supplemented with 10% fetal calf serum, 100 U/mL penicillin and 100 µg/mL streptomycin. Cells were grown at 37 °C and 5% CO_2_. Functionality of the cells was confirmed by measuring uptake of prototypic substrates (see Section 2.3 and supplementary material).

### 2.3. Cellular Uptake Studies

Uptake assays were conducted at 37 °C according to previously described methods [13,18,19,20]. For this, cells were seeded in 24-well plates (320,000 cells/well) and cultured for 2 days. Cells were then incubated for a further 24 h in culture medium with 5 mM butyrate (OATP1B3, OATP2B1, OCT1) or 10 mM butyrate (OATP1B1) to increase protein levels of recombinant transporters. Before the uptake experiments, cells were washed with the respective pre-warmed (37 °C) uptake buffer. The compositions of the uptake buffers were as follows: for OATP1B1, OATP1B3, OATP2B1: 142 mM NaCl, 12.5 mM hydroxyethylpiperazine ethanesulfonic acid, 5 mM KCl, 1 mM KH_2_PO_4_, 1.5 mM CaCl_2_, 1.2 mM MgSO_4_, 5 mM glucose, pH 7.3; for OCT1: 145 mM NaCl, 5 mM hydroxyethylpiperazine ethanesulfonic acid, 3 mM KCl, 1 mM CaCl_2_, 0.5 mM MgCl_2_, 5 mM glucose, pH 7.4. Uptake was initiated by replacing this solution with uptake buffer containing different concentrations of remdesivir as indicated. Uptake was carried out at 37 °C and stopped after 10 min by removal of the uptake solution and then washing the cells twice with ice-cold uptake buffer and twice with ice-cold phosphate-buffered saline. Plates were frozen at −20 °C until lysis. For cell lysis, plates were thawed on ice and cells were harvested by scraping them off in 250 µL acetonitrile/H_2_O 1:1 (*v/v*) (Carl Roth, Karlsruhe, Germany) containing 0.1% formic acid and transferring them into Eppendorf tubes. The lysis buffer contained QX as internal standard at a final concentration of 0.5 µM. Cells were disrupted by three cycles of shock freezing/thawing (liquid nitrogen, 37uC water bath) and further by ultra-sonification, three times 3 sec, at 4 °C. Disrupted cell solutions were centrifuged 5 min (15,000 *g*) at 4 °C and supernatants were transferred into Eppendorf tubes and stored at −20 °C for analytic determination of remdesivir using ultra-high-performance liquid chromatography-tandem mass spectrometry (UHPLC-MS-MS).

Prototypic substrate uptake was performed as described previously. For this, uptake solutions contained the respective substrate with tracer amounts of radiolabeled substrate. In case of inhibition studies, uptake solutions additionally contained different concentrations of remdesivir. Cells were incubated at 37 °C with the uptake solutions and uptake was stopped by removal of the uptake solutions and washing the cells three times with ice-cold uptake buffer. Cells were then lysed with 0.2% sodium dodecyl sulfate and intracellular radioactivity was measured by liquid scintillation counting (Tricarb2800, PerkinElmer Life Sciences GmbH, Rodgau, Germany or Hidex 300SL TDCR liquid scintillation counter, Turku, Finland). The following conditions were used: final concentration of 0.05 µM BSP and 10 min uptake for OATP1B1 [21], 5 µM estradiol glucuronide and 5 min uptake for OATP1B3 [19], 1 µM estrone sulfate and 30 sec uptake for OATP2B1 [13] and 5 µM metformin and 5 min uptake for OCT1 [20].

Protein contents of the lysed cells were determined with the bicinchoninic acid (BCA) assay according to Smith et al. [22] and as described [13,18,19,20]. In brief, the BCA reagent was prepared by mixing a 4% copper II sulfate solution with BCA solution at a 1:50 ratio. Standards were prepared through dilution of bovine serum albumin (1 mg/mL) with water to obtain concentrations of 200, 400, 600 or 800 μg/mL. 10 μL of standards or samples were mixed with 200 μL BCA reagent and incubated for 1 h at 37 °C. The absorbance of the samples and standards was then measured at 570 nm. The total protein content of the samples was calculated relative to the protein content of the standards.

Cell viability was not determined because it is not expected that the short term incubations with remdesivir of less than 10 min will affect viability.

### 2.4. Quantification of Remdesivir by UHPLC-MS-MS

Remdesivir was determined by UHPLC-MS-MS analysis on an Agilent 1290 infinity UHPLC system coupled to an Agilent 6495B triple quadrupole mass spectrometer (Agilent, Waldbronn, Germany) similar to a recently published method [23]. QX was used as internal standard. Ionization mode was electrospray (ESI), polarity positive with the following conditions: capillary voltage 3500 V, nozzle voltage 1000 V, drying gas flow 11 l/min nitrogen, drying gas temperature 225 °C, nebulizer pressure 20 psi, sheath gas temperature 350 °C, sheath gas flow 11 l/min.

The analytes were separated on an Acquity HSS T3 column (100 mm × 2.1 mm I.D., 1.8 µm particle size; Waters, Eschborn, Germany) using (A) 0.1% (*v/v*) formic acid in water and (B) 0.1% (*v/v*) formic acid in acetonitrile as mobile phases. The gradient started at 10% B for 0.30 min, increased to 20% B to 0.35 min, then to 70% B to 1.5 min, and to 90% B to 1.8 min, remained at 90% B until 3.8 min, followed by re-equilibration to 10 % B for 1.7 min. The flow rate was 0.4 mL/min, and the injection volume was 0.5 µL. The mass spectrometer was operated in the multiple reaction monitoring (MRM) mode, using the transitions, *m*/*z* 603.2 > 228.9 for remdesivir and *m*/*z* 313.2 > 247.1 for QX. Dwell time was 100 ms. Fragmentor voltage was set at 380, and the collision energy at 32 and 40 V for remdesivir and QX, respectively.

Remdesivir calibration samples were prepared in water: acetonitrile 1:1 (*v/v*) containing 0.1% formic acid and 0.5 µM internal standard, in a concentration range of 5 nM to 10 µM. Calibration samples were analyzed together with the unknown samples. Calibration curves based on internal standard calibration were obtained by weighted (1/x) linear regression for the peak area ratio of the analyte to the internal standard against the amount of the analyte. The concentration in unknown samples was obtained from the regression line. Assay accuracy and precision were determined by quality controls that were prepared like the calibration samples. Data on method validation are summarized in the supplementary material.

### 2.5. Statistical Analysis

Transport data were analyzed with GraphPad Prism 9.0.2 (GraphPad Software Inc., La Jolla, CA, USA) and are means ± SEM. The Brown-Forsythe/Welch ANOVA test followed by a Dunnett’s T3 posthoc test as multiple comparisons test were performed to determine statistical significance in all experiments. Statistical tests were two-tailed and *p* values < 0.05 were considered significant.

## 3. Results

### 3.1. Assessment of Remdesivir as Transported Substrate of Hepatic Uptake Transporters

**Figure 1:**
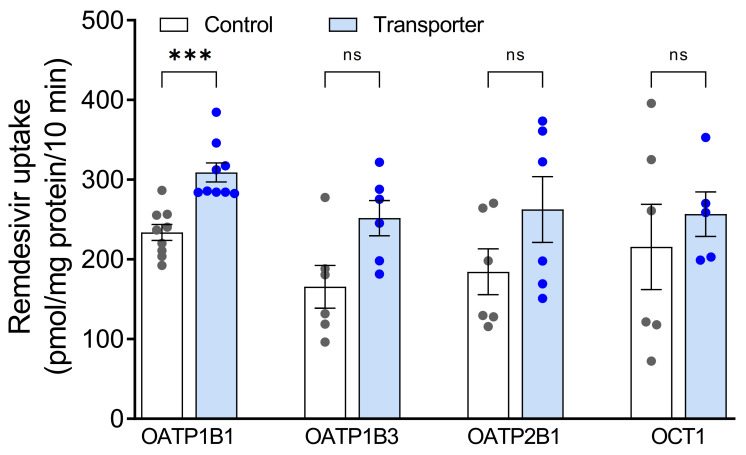
Accumulation of remdesivir (5 µM) after 10 min in HEK cells stably expressing hepatic uptake transporters OATP1B1, OATP1B3, OATP2B1, OCT1 (filled bars) or vector-transfected control cells (open bars). Data are individual values with means ± SEM of 5-9 wells. *** *p* < 0.001; ns, not significant.

At first, accumulation of 5 µM remdesivir into cells expressing OATP1B1, OATP1B3, OATP2B1 or OCT1 was investigated. This is a relevant clinical concentration because the C_max_ value was 3.7 µM after intravenous administration of clinically-used doses of 100 mg remdesivir to healthy adults [4]. A high remdesivir accumulation into the respective control cells was already detected (Figure 1). Remdesivir uptake ratios were 1.3, 1.5, 1.4 and 1.2 for OATP1B1, OATP1B3, OATP2B1 and OCT1, respectively. Statistically significant uptake was only detected for OATP1B1.

**Figure 2:**
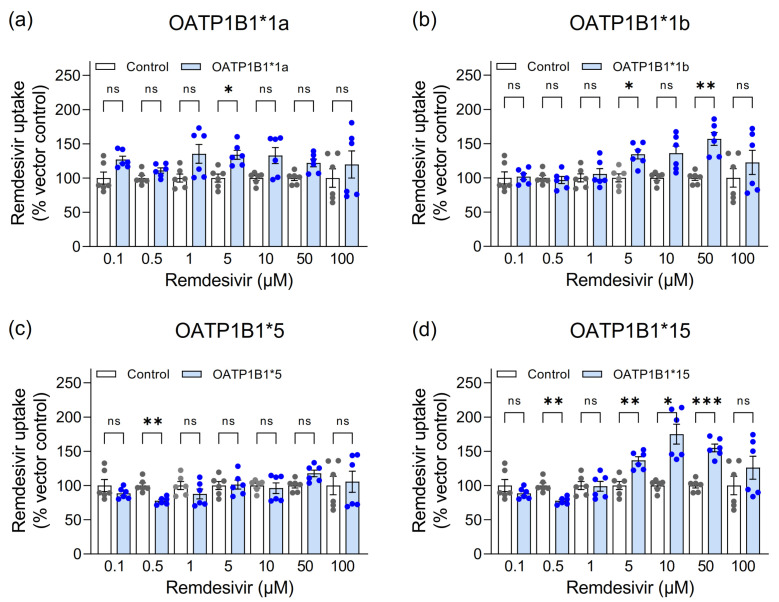
Accumulation of remdesivir (0.1 to 100 µM) after 10 min into HEK cells stably expressing OATP1B1*1a (**a**), OATP1B1*1b (**b**), OATP1B1*5 (**c**) and OATP1B1*15 (**d**) and vector-transfected control cells (white bars). Data are individual values with means ± SEM of 6 wells. * *p* < 0.05; ** *p* < 0.01; *** *p* < 0.001; ns, not significant.

As statistically significant uptake was only detected for OATP1B1 and OATP1B1 has been reported as remdesivir transporter [4] and genetic variants may affect the OATP1B1-mediated transport [18], we pursued functional characterization of OATP1B1 and its common genetic variants *1b (OATP1B1p.N130D), *5 (p.V174A) and *15 (p.N130D+V174A). Again, cellular remdesivir accumulation was largely independent of OATP1B1. At a concentration of 5 µM, remdesivir accumulated to a moderately higher extent into OATP1B1*1a-, OATP1B1*1b- and OATP1B1*15-expressing cells than into vector-transfected control cells (Figure 2). Accumulation was also moderately higher into OATP1B1*1b cells at 50 µM and into OATP1B1*15 cells at 10 and 50 µM.

### 3.2. Assessment of Remdesivir as Inhibitor of Hepatic Uptake Transporters

**Figure 3:**
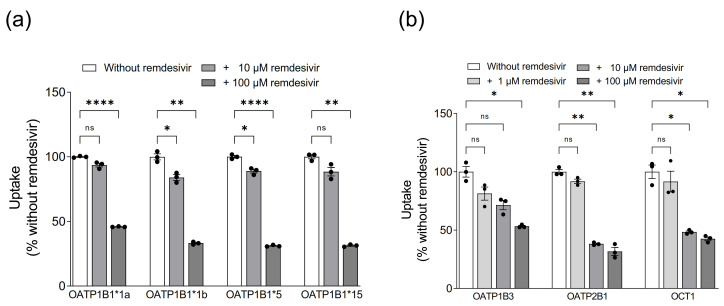
Inhibition of OATP1B1- (**a**), and OATP1B3-, OATP2B1- and OCT1-mediated (**b**) transport by remdesivir. Data are shown as the percentage of prototypic substrate uptake (OATP1B1: BSP; OATP1B3: estradiol glucuronide; OATP2B1: estrone sulfate; OCT1: metformin) in the absence of remdesivir. Data are individual values with means ± SEM of 3 wells. * *p* < 0.05; ** *p* < 0.01; **** *p* < 0.0001; ns, not significant.

While the low uptake rates suggested that OATP1B1, OATP1B3, OATP2B1 and OCT1 are not relevant for hepatocellular remdesivir uptake, remdesivir might still interact with these transporters as inhibitor. We therefore investigated whether remdesivir inhibits the uptake of prototypic transporter substrates. Remdesivir had a significant concentration-dependent inhibitory effect on all investigated transporters (Figure 3).

## 4. Discussion

Remdesivir is the first FDA-approved antiviral agent for treatment of hospitalized COVID-19 patients [3]. It experiences a high first pass effect indicating rapid uptake into the liver. Accumulation into the liver may reduce remdesivir plasma concentrations with an impact on efficacy. As membrane transporters are known to mediate hepatocellular drug uptake, it was the goal of this in vitro study to investigate whether the major hepatic uptake transporters OATP1B1, OATP1B3, OATP2B1 and OCT1 are involved in remdesivir uptake. A second aim was to assess remdesivir’s potential to inhibit these transporters.

Our data of high remdesivir uptake already into control cells and low uptake ratios at a clinically relevant drug concentration suggest that neither OATP1B1, OATP1B3, OATP2B1 nor OCT1 are important for hepatocellular remdesivir uptake in humans. Al-though the information given in the package insert of Veklury identifies OATP1B1 as transporter of remdesivir [4] and our data show a statistically significant higher uptake into OATP1B1-expressing cells compared with control cells, the uptake ratio is only 1.3. This is considerably less than the recommended 2-fold ratio which should be achieved for considering a compound a transported substrate [24,25].

We also analyzed the effect of the different OATP1B1 genetic variants OATP1B1*1b, OATP1B1*5 and OATP1B1*15 on remdesivir uptake because these had been shown to impact on the cellular uptake of clinically-used drugs, such as statins, with consequences on pharmacokinetics and drug effects [8,12,13]. Despite significantly higher uptake ratios for OATP1B1*1b and OATP1B1*15 for some concentrations, uptake ratios were again low indicating that these genetic variants also do not impact on hepatocellular remdesivir uptake either. The significantly lower uptake by OATP1B1*5 and OATP1B1*15 at a concentration of 0.5 µM remdesivir will presumably also not impact hepatocellular uptake due to the high OATP1B1-independent uptake.

As OATP1B1, OATP1B3, OATP2B1 and OCT1 are known to mediate drug-drug interactions [7,9,11,12], we also evaluated remdesivir as inhibitor of these transporters. We observed inhibition of all investigated transporters already at a concentration of 10 µM. This concentration is close to the peak plasma concentration observed 30 min after iv administration of 200 mg remdesivir for 30 min [26]. Interaction of remdesivir with OATP1B1 and OATP1B3 had been described in the compassionate use authorization issued by EMA [27], which reports IC_50_ values of 2.8 µM and 2.1 µM, respectively, however without stating the prototypic substrates used for the experiments. Recently, inhibition of OATP2B1-mediated pyranine transport with an IC_50_ value of 3.8 µM was reported [28]. Our data support these observations and extend inhibition data to OCT1 as well. Since remdesivir is very rapidly cleared (t_1/2_ = 1 h) [26], its potential to be a perpetrator of clinically-significant drug-drug interactions mediated by OATP1B1, OATP1B3, OATP2B1 and OCT1, however, is considered to be limited [27].

In conclusion, this is the first report on interaction of remdesivir with the major hepatic drug uptake transporters OATP1B1, OATP1B3, OATP2B1 and OCT1. Our data indicate that these transporters do not play a crucial role in hepatocellular uptake of remdesivir or in drug-drug interactions mediated by these transporters.

## Data Availability

Data is contained within the article.

## Supplementary Materials

The following are available online at https://www.mdpi.com/1999-4923/13/3/369/s1, Table S1: Intra- and inter-day variability of remdesivir in cellular extracts, Table S2: Recovery and matrix effect of remdesivir, Figure S1: Prototypic substrate uptake into cells expressing the investigated transporters (blue bars) in comparison to uptake into vector-transfected control cells (white bars). The following conditions were used: final concentration of 0.05 µM BSP and 10 min uptake for OATP1B1 [21] and OATP1B1 variants, 5 µM estradiol glucuronide and 5 min uptake for OATP1B3 [19], 1 µM estrone sulfate and 30 sec uptake for OATP2B1 [13] and 5 µM metformin and 5 min uptake for OCT1 [20]. Data are individual values with means ± SEM of 3 wells. ** *p* < 0.01; **** *p* < 0.0001.
