# Supplementary material for: Interaction of Remdesivir with Clinically Relevant Hepatic Drug Uptake Transporters
Source: Pharmaceutics. 2021 Mar 10;13(3):369. doi: 10.3390/pharmaceutics13030369 (PMC7999182; doi:10.3390/pharmaceutics13030369)
Supplement: Supplementary file 1 [file pharmaceutics-13-00369-s001.pdf]

pharmaceutics

# Supplementary Materials: Interaction of Remdesivir with Clinically Relevant Hepatic Drug Uptake Transporters

Anne T. Nies , Jörg König , Ute Hofmann , Charlotte Kölz , Martin F. Fromm and Matthias Schwab

## Method validation

*Accuracy and precision*

Assay accuracy and precision were determined by quality controls that were prepared like the calibration samples.

**Table S1.** Intra- and inter-day variability of remdesivir in cellular extracts.

| Quality control | Conc. | Intra (n = 6) | | Inter (n = 15) | |
|---|---|---|---|---|---|
| | [nM] | Dev.[a] [%] | CV [b] [%] | Dev. [%] | CV [%] |
| LLOQ | 5 | 10.0 | 9.6 | -4.4 | 15.3 |
| low | 10 | 2.6 | 3.0 | 0.2 | 7.3 |
| medium | 100 | 6.9 | 2.9 | 0.4 | 4.8 |
| high | 1000 | 10.9 | 1.4 | 2.4 | 8.0 |

[a] Dev. = deviation of the mean from the concentration added

[b] CV = mean relative standard deviation

**Publisher's Note:** MDPI stays neutral with regard to jurisdictional claims in published maps and institutional affiliations.

**Copyright:** © 2021 by the authors. Submitted for possible open access publication under the terms and conditions of the Creative Commons Attribution (CC BY) license (http://creativecommons.org/licenses/by/4.0/).

## Recovery and matrix effect

The recovery was determined by comparing peak areas from diluted standard solutions to peak areas of the same concentration in blank cell lysates. The internal standard normalized matrix effect was determined by comparing the area ratio of remdesivir to the internal standard in diluted standard solutions to the area ratio in blank cell lysates. Recovery and matrix effect were determined over the whole concentration range measured.

**Table S2.** Recovery and matrix effect of remdesivir.

| | Recovery remdesivir [%] | Recovery ISTD [%] | Matrix effect remdesivir [%] |
|---|---|---|---|
| Mean (n=7) | 86.1 | 90.5 | 95.3 |
| CV | 5.5 | 6.7 | 3.1 |

**Stability**

Sample stability was evaluated for the intended storage time.

Samples were stable up to 24 h in the autosampler compartment, repeated analysis showed deviations between -6.9 % and 6.2 %.

**Control uptakes**

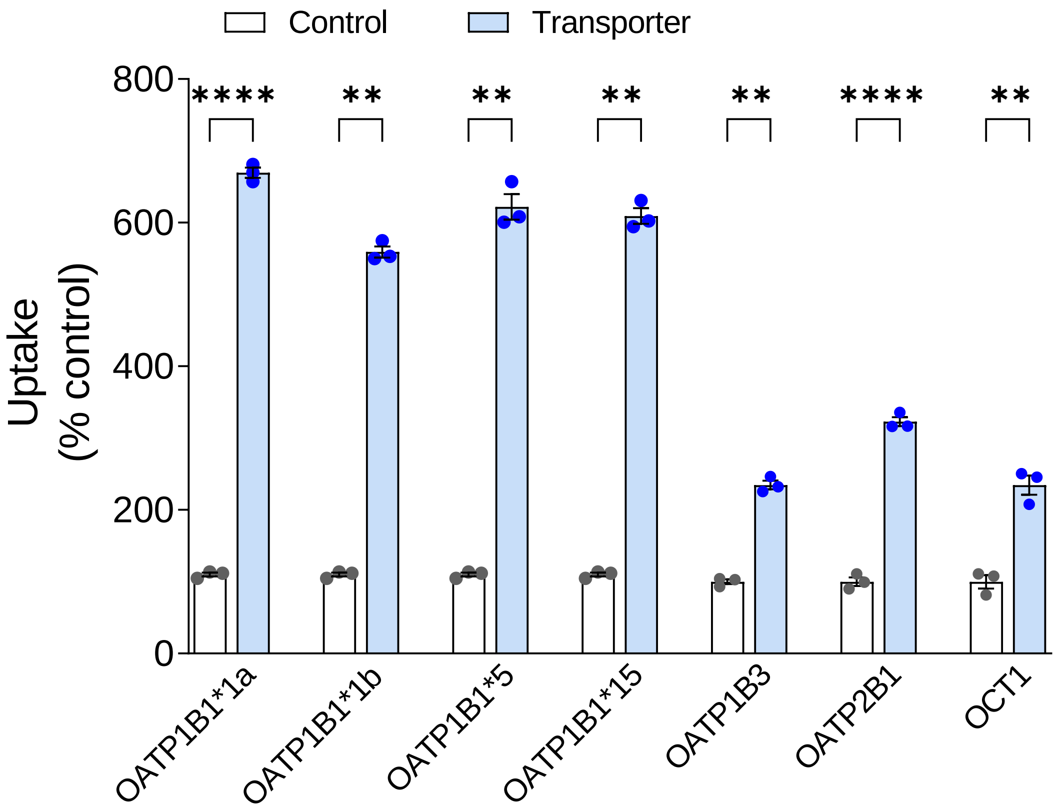


**Figure S1.** Prototypic substrate uptake into cells expressing the investigated transporters (blue bars) in comparison to uptake into vector-transfected control cells (white bars). The following conditions were used: final concentration of 0.05 µM BSP and 10 min uptake for OATP1B1 [21] and OATP1B1 variants, 5 µM estradiol glucuronide and 5 min uptake for OATP1B3 [19], 1 µM estrone sulfate and 30 sec uptake for OATP2B1 [13] and 5 µM metformin and 5 min uptake for OCT1 [20]. Data are individual values with means ± SEM of 3 wells. ** $p < 0.01$; **** $p < 0.0001$.
